# Prevalence, concordance and determinants of human papillomavirus infection among heterosexual partners in a rural region in central Mexico

**DOI:** 10.1186/1471-2334-10-223

**Published:** 2010-07-28

**Authors:** Rocio Parada, Rosalba Morales, Anna R Giuliano, Aurelio Cruz, Xavier Castellsagué, Eduardo Lazcano-Ponce

**Affiliations:** 1 Centro de Investigación en Salud Poblacional, Instituto Nacional de Salud Pública, Cuernavaca, Morelos, México; 2 Instituto Mexicano del Seguro Social, Morelos, México; 3 H. Lee Moffitt Cancer Center and Research Instutute, Tampa, Florida; 4 Cancer Epidemiology Research Program, Institut Català d´Oncologia (ICO), IDIBELL, CIBER-ESP, L’Hospitalet de Llobregat, Spain

## Abstract

**Background:**

Although human papillomavirus (HPV) infection in heterosexual couples has been sparsely studied, it is relevant to understand disease burden and transmission mechanisms. The present study determined the prevalence and concordance of type-specific HPV infection as well as the determinants of infection in heterosexual couples in a rural area of Mexico.

**Methods:**

A cross-sectional study was conducted in 504 clinically healthy heterosexual couples from four municipalities in the State of Mexico, Mexico. HPV testing was performed using biotinylated L1 consensus primers and reverse line blot in cervical samples from women and in genital samples from men. Thirty-seven HPV types were detected, including high-risk oncogenic types and low-risk types. Multivariate logistic regression models were utilized to evaluate factors associated with HPV.

**Results:**

The prevalence of HPV infection was 20.5% in external male genitals and 13.7% in cervical samples. In 504 sexual couples participating in the study, concordance of HPV status was 79%; 34 partners (6.7%) were concurrently infected, and 21 out of 34 partners where both were HPV positive (61.8%) showed concordance for one or more HPV types. The principal risk factor associated with HPV DNA detection in men as well as women was the presence of HPV DNA in the respective regular sexual partner (OR = 5.15, 95%CI 3.01-8.82). In men, having a history of 10 or more sexual partners over their lifetime (OR 2.5, 95%CI 1.3 - 4.8) and having had sexual relations with prostitutes (OR 1.7, 95%CI 1.01 - 2.8) increased the likelihood of detecting HPV DNA.

**Conclusions:**

In heterosexual couples in rural regions in Mexico, the prevalence of HPV infection and type-specific concordance is high. High-risk sexual behaviors are strong determinants of HPV infection in men.

## Background

Although there is clear evidence for the influence of the male factor in the development of cervical neoplasia[1,2], HPV transmission in heterosexual couples has rarely been studied. The few studies conducted have included the male sexual partners of women with clinical HPV lesions [3-8] In addition, heterosexual couples have been studied through controlled clinical trials to evaluate the effect of the use of condoms on the rate of persistence of flat penile lesions[9]. Previous reports from prospective studies of women initiating sexual life have estimated an accumulated HPV risk of 50% over a period of three years. The risk of HPV infection in these women increases if the male sexual partner had initiated sexual life at an early age[10]. In light of the scarce studies exploring HPV transmission among heterosexual couples, mathematical models have emerged to simulate HPV transmission dynamics. A greater transmissibility of HPV has been estimated as compared to other sexually transmitted infections, such as HIV and type 2 herpes simplex[11]. Information about HPV transmission probabilities in couples is of paramount importance to evaluate the impact of prophylactic vaccines against HPV and to monitor the distribution of specific types before and after the introduction of HPV vaccines.

The goal of the present study was to determine genital HPV prevalence in sexual couples, evaluate HPV type-specific concordance and the association of known risk factors of HPV infection in a low-risk, predominantly monogamous population.

## Methods

We conducted an HPV DNA prevalence and type-specific concordance study in 504 heterosexual couples attending first-level health centers for medical attention in four municipalities belonging to the Texcoco Sanitary District in the State of Mexico, in the central region of the Mexican Republic. Three of the municipalities had rural characteristics (Atenco, Tepetlauxtoc, Texcoco) and one was semi-urban (Chimalhuacan). The study period was November 2002 to September 2003.

The study partners were identified and selected using convenience sampling in healthcare centers. First, women who consistently sought care in health centers for diverse reasons were identified and invited to participate. The women who were accepted into the study were asked to invite their regular sexual partner to participate (having been a sexual couple for six or more months even if they were not living in the same house). Subjects were invited to participate after a talk about HPV infection and its association with cervical cancer given at the participating healthcare centers. Since the professional occupation for 65% of this population is agriculture, the study was conducted during the morning hours to increase the likelihood that the male sexual partner would attend.

Sexual couples were included when both partners were available to participate in the study; excluded were sexual couples in which the female partner was pregnant or had a hysterectomy.

Male partners were instructed not to wash their genitals for at least 12 hours prior to the examination and to be sexually abstinent for three days. Female partners were asked to be sexually abstinent and not menstruating.

After receiving written informed consent guaranteeing confidentiality, and in complete privacy, a self-administered questionnaire was completed to obtain information about socioeconomic variables, educational level, smoking habits, reproductive history, use of contraceptive methods and sexual behavior factors. The couples answered the questionnaire in separate locations. The questionnaires and collection of biological samples for each partner were carried out on the same day at the corresponding health center. The study was approved by the ethical and research committees at the institutions that participated in the study. The overall response rate was 60%.

### Collection of specimens

The methods used to collect the samples from the male genital area have previously been described[12]. Briefly, epithelial cells from three anatomic sites were obtained using a cytobrush and a Dacron swab: The first sample was obtained from the scrotum and the penile shaft, the second from the balano-preputial lamina, and the third from the urinary meatus. The three samples were combined into one single tube and stored.

In women, a sample of epithelial cells was taken from the exocervix and endocervical canal using a nylon cytobrush, which was rotated 360°C to assure sampling of the cervical transformation zone. All genital samples were collected by a trained doctor. All brushes containing the collected material were placed in a 5 ml aliquot of phosphate-buffered saline (PBS)/merthiolate 0.01% (v/v). The samples were maintained at -20°C for an average of 30 days, until their delivery to the laboratory. The samples were also stored at -20°C in the laboratory prior to DNA extraction.

### DNA extraction

Previous to DNA extraction all samples which arrived at the lab were centrifuged at 4500 rpm for 6 minutes, after pellet was suspended in 1 ml of 0.01 M TRIS HCL pH 7.4. Briefly, genital samples were treated with proteinase K (170 ug/ml). The DNA extraction was performed with phenol-chlorophorm/isoamyl alcohol 24:1, then NaCl 5 M was added and precipitated with isopropanol. Finally pellet was suspended in 50 μl of buffer TE pH 7.6 and stored at -70°C [13].

### HPV DNA amplification, detection and genotyping

#### HPV DNA amplification

Was performed using DNA hybridization test as described by Gravitt et al [14]. After sample extraction amplification of HPV DNA and β-globin was conducted in separate reactions. HPV DNA was amplified using biotinylated PGMY L1 consensus primer. To determine specimen adequacy, a fragment of the human β-globin gene was co-amplified with primers BGH20 and BPC04.

#### HPV DNA detection and genotyping

HPV detection and genotyping was performed on the products of PCR (inverse hybridization) which utilized the nylon membranes that were used for the hybridization. Each membrane contained 39 test lines, 37 of which correspond to type-specific HPV and two to the quantification of low and high concentrations of β-globin. HPV types that were considered high-risk for the development of cervical neoplasia are 16, 18, 31, 33, 35, 39, 45, 51, 52, 56, 58, 59, 66; low-risk are 6, 11, 26, 40, 42, 53, 54, 55, 61, 62, 64, 67, 68, 69, 70, 71, 72, 73, 81, 82, 83, 84, IS39, CP6108[15]. The hybridization bands were detected using colorimetry. Membranes were interpreted using acetate that indicated the position of each HPV type. Hybridization results were independently interpreted by two reviewers. In addition, for the purpose of analysis, β-globin-negative samples that were PCR positive to HPV were considered as positive, given that competition between oligoelements from the diagnostic strips could result in a β-globin-negative specimen.

### Statistical Analysis

A stratified analysis was conducted by sex and a socioeconomic level index (SLI) was created using principal component analysis with the variables education, floor material in the dwelling, availability of potable water, drainage availability, owning a vehicle and owning domestic electronic equipment such as a television, video cassette recorder, gas stove and water heater. The index obtained was categorized in tertiles to define low, medium, and high SLI. The McNemar's test was used to compare the prevalence of HPV among men and their sexual partners.

The concordance of HPV status between sexual partners and between HPV risk groups (high- and low-risk) was evaluated based on contingency tables using the Kappa statistic. Concurrence of HPV infection in heterosexual couples was defined as the presence of infection in both of the sexual partners, independently of whether or not there was HPV type concordance. For couples where both partners presented with at least one type of HPV infection, percentages for type-specific positive concordance were obtained and, for those with type-specific concordance, it was determined whether the partners were concordant for one type, two types, or three types. The association of potential determinants of HPV positivity in both men and women was evaluated using logistic regression modeling adjusted for age and SLI, obtaining odds ratios (OR) with 95% confidence intervals. All p-values were two-sided.

## Results and Discussion

### Prevalence and concordance of HPV infection in sexual couples

**Figure 1:**
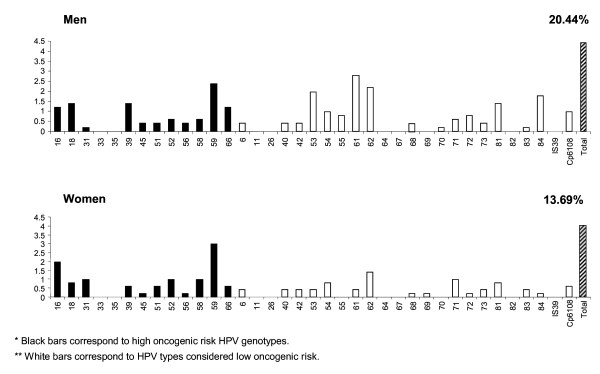
**Type specific prevalence of HPV infection in a group of heterosexual couples in central Mexico, according to sex**.

The prevalence of HPV infection was 20.4% in men and 13.7% in women. The most frequently detected high-risk types were HPVs 59, 18, 39 and 16 among men, and HPVs 59, 16, 31, 52 and 58 among women. The most common low-risk types were HPVs 61, 62, 53, 84 and 81 among men, and HPVs 62, 71, 81 and 54 among women. Overall, the pattern of HPV type distribution was similar among men and women (Table 1 and Figure 1).

**Table 1:** Prevalence of HPV DNA in 504 heterosexual couples in central Mexico, according to sex.

	Men n = 504		Women n = 504		
		
HPV	n	%	n	%	ρ*
**Presence of HPV**					
Positive	103	20.44	69	13.69	0.0009
**Presence of high-risk HPV**					
Positive	44	8.73	48	9.52	0.6056
**Presence of low-risk HPV**					
Positive	75	14.88	33	6.55	0.000
**Multiple HPV infection**					
One type only	79	15.67	50	9.92	0.3841
Two or more types	24	4.77	19	3.77	
**Presence of HPV 16 and/or 18**					
Negative	491	97.42	490	97.22	0.8348
Positive	13	2.58	14	2.78	
					
**Positive for High-risk HPV**					
16	6	1.19	10	1.98	0.2850
18	7	1.39	4	0.79	0.3173
31	1	0.20	5	0.99	0.0455
33	0	0	0	0	
35	0	0	0	0	
39	7	1.39	3	0.60	0.1025
45	2	0.40	1	0.20	0.5637
51	2	0.40	3	0.60	0.6547
52	3	0.60	5	0.99	0.4142
56	2	0.40	1	0.20	0.3173
58	3	0.60	5	0.99	0.4142
59	12	2.38	15	2.98	0.4913
66	6	1.19	3	0.60	0.2568
					
**For low-risk HPV**					
6	2	0.40	2	0.40	1.000
11	0	0	0	0	
26	0	0	0	0	
40	2	0.40	2	0.40	1.000
42	2	0.40	2	0.40	1.000
53	10	1.98	2	0.40	0.0114
54	5	0.99	4	0.79	0.6547
55	4	0.79	0	0	0.045
61	14	2.78	2	0.40	0.0013
62	11	2.18	7	1.39	0.2059
64	0	0	0	0	
67	0	0	0	0	
68	2	0.40	1	0.20	0.5637
69	0	0	1	0.20	0.3173
70	1	0.20	0	0	0.3171
71	3	0.60	5	0.99	0.4142
72	4	0.79	1	0.20	0.1797
73	2	0.40	2	0.40	1.000
81	7	1.39	4	0.79	0.3173
82	0	0	0	0	
83	1	0.20	2	0.40	0.5637
84	9	1.79	1	0.20	0.0047
IS39	0	0	0	0	
Cp6108	5	0.99	3	0.60	0.4142


* p-value obtained using  McNemar´s Test.

Concordance of HPV status was 79%. In 138 couples of the 504 included in the study, at least one of the respective partners had some type of HPV infection (27.4%). In 69(50%) of these only the man presented some type of infection, in 35(25.4%) only the woman, and in 34(24.6%) both were infected. Among heterosexual couples in which both partners were infected, 21(61.8%) showed type-specific concordance in one or more HPV types. Overall concordance was statistically significant (Kappa = 0.28, p < 0.001). The most frequent HPV types found in both partners who presented type-specific concordant infection were HPVs 59, 62, 54 and 39 (Table 2).

**Table 2:** Group and type-specific HPV concordance in 504 heterosexual couples

		Female partner			
	
	Negative	high-risk HPV	low-risk HPV	HPV Both types:	Total
Male partner					
Negative	366	20	10	5	401
high-risk HPV	19	**5**	**2**	**2**	28
low-risk HPV	45	**6**	**9**	**3**	63
HPV, both types	5	**3**	**1**	**3**	12
Total	435	34	22	13	504


**Type-specific concordance of HPV infection^a ^in 34 sexual couples with concurrent infection**.					

		n		%	

No concordance		13		38.24	
Concordance of at least one type		21		61.76	

**HPV genotypes detected in 34 sexual couples, both with infection**.					

**No**.	**Male genitals' HPV type ^b^**	**Cervical HPV type**			

1	53	52			
2	84	84			
3	39,53	39,53,81			
4	51,56	56			
5	61	39			
6	62	62			
7	59	59			
8	81	6			
9	62,66, cp6108	31,40,62,66,72, cp6108			
10	18,81	18,81			
11	6	6			
12	16,39,84	54			
13	16	16			
14	54	54			
15	52,58,61,71,81	52,58,73			
16	39	39,71			
17	72	53			
18	55	52,58			
19	59	59			
20	55	18			
21	62,84	16,62			
22	59,62	16			
23	71,73	71			
24	16,59	59			
25	58	66			
26	66	16			
27	18,39,62,66	58			
28	54	54			
29	68	71,83			
30	61	31,61			
31	31	31,81			
32	40	59			
33	59,84	59			
34	62	62,71			

Concordance of HPV status observed: 79% K = 0.28, p value < 0.001.

Note: Concordance of status corresponds to the percentage of couples in which both partners were HPV negative or both were positive, independently of risk group or HPV type. Concurrent infection corresponds to the percentage of couples for which both partners were positive independently of the risk group or HPV type. Number in bold indicates couples with concurrent infection (34).

^a ^When infection is present in both men and women and there is at least one HPV genotype in common.

^b ^Sample taken from the combination of epitheleal cells from the middle third of the scrotum and penile shaft, the balano-preputial lamina and the urinary meatus.

### Determinants of HPV infection

#### Sociodemographic characteristics

In the study population, 69.4% of the couples lived in a rural area, 79.4% were married or living together, and 85.3% were Catholic. The median age for men was 38 years old and for women, 35 years. Seventy-four percent of men and 73% of women had nine or less than nine years of schooling. Forty-five percent of the men and 14% of the women were current smokers. A multiple logistic regression analysis, adjusted for age and SLI, indicated that among men, living in an urban area was significantly associated with an increased risk of penile HPV infection (OR 1.7, 95%CI 1.1 - 2.7) compared to living in a rural area. In addition, being single (OR 1.9, 95%CI 1.1 - 3.2) and having less than 7 years of schooling (OR 1.8, 95%CI 1.0 - 3.4) were variables significantly associated with an increased risk for penile HPV infection. For women, not having a stable partner was associated with a statistically significant increase in the risk of cervical HPV infection (OR 2.8, 95%CI 1.6 - 5.0), as was being a current smoker (OR 2.0, 95%CI 1.03 - 3.7). Age, years of schooling, SLI, and religion were not associated with the presence of cervical HPV infection in women (Table 3).

**Table 3:** Sociodemographic and sexual conduct characteristics associated with the presence of HPV DNA among 504 heterosexual couples in central Mexico, according to sex.

	Men n = 504^a^				Women n = 504^a^			
Variable		HPV + n = 103	Risk of HPV infection			HPV + n = 69	Risk of HPV infection	
	**n (%)**	**HPV + (%)**	**OR^b^**	**CI 95%**	**n (%)**	**HPV + (%)**	**OR^b^**	**CI 95%**

**Age^c ^(years)**								
18-24	40(8)	9(22.50)	1.0		64(12.7)	13(20.31)	1.0	
25-30	91(18)	17(18.68)	.77	.31 - 1.93	98(19.4)	15(15.31)	.70	.30 - 1.60
31-40	191(37.9)	29(15.18)	.61	.26 - 1.42	209(41.5)	24(11.48)	.47	.22 - 1.00
41-75	182(36.1)	48(26.37)	1.23	.54 - 2.80	133(26.4)	17(12.78)	.55	.24 - 1.23
*p-trend*				0.1999				0.1305
**Place of residence**								
Rural	350(69.4)	62(17.71)	1.0		350(69.4)	47(13.43)	1.0	
Urban	154(30.6)	41(26.62)	1.71	1.08 - 2.71	154(30.6)	22(14.29)	1.02	.58 - 1.79
**Marital Status**								
Married	400(79.4)	72(18)	1.0		400(79.4)	43(10.75)	1.0	
Single	104(20.6)	31(29.81)	1.92	1.14 - 3.25	104(20.6)	26(25.00)	2.79	1.56 - 5.00
**Schooling^d^**								
<= 6 years	174(34.5)	47(27)	1.85	.99 - 3.44	77(15.5)	8(10.39)	.70	.28 - 1.76
7-9 years	199(39.5)	37(18.6)	1.28	.70 - 2.36	286(57.6)	43(15.03)	1.17	.62- 2.19
>= 10 years	131(26)	19(14.5)	1.0		134(26.9)	17(12.69)	1.0	
*p-trend*				0.0061				0.8069
**Religion**								
Catholic	430(85.3)	81(18.84)	1.0		430(85.3)	58(13.49)	1.0	
Other	74(14.7)	22(29.73)	1.88	1.07 - 3.31	74(14.7)	11(14.86)	1.04	.51 - 2.11
**Current smoker**								
No	278(55.2)	56(20.14)	1.0		435(86.3)	53(12.18)	1.0	
Yes	226(44.8)	47(20.80)	1.08	.69 - 1.69	69(13.7)	16(23.19)	1.97	1.03 - 3.75
**Age on initiating sexual life**								
≤18 years	284(56.35)	68(23.94)	1.59	1.003 - 2.52	269(53.4)	39(14.50)	1.06	.62 - 1.81
≥19 years	220(43.65)	35(15.91)	1.0		235(46.6)	30(12.77)	1.0	
**No. of lifetime sexual ** **partners**								
One	185(36.7)	30(16.22)	1.0		371(73.6)	45(12.13)	1.0	
Two	76(15.1)	17(22.37)	1.49	.75 - 2.92	88(17.5)	15(17.05)	1.50	.78 - 2.85
Three to nine	171(33.9)	31(18.13)	1.08	.62 - 1.90	45(8.9)	9(20.00)	1.69	.75 - 3.79
Ten or more	72(14.3)	25(34.72)	2.54	1.34 - 4.82	--	--	--	--
*P-trend*				0.0142				0.0796
**History of anal sexual ** **prelations**								
No	305(63.15)	64(20.98)	1.0		146(67)	25(17.12)	1.0	
Yes	178(36.85)	34(19.10)	.90	.56 - 1.45	72(33)	8(11.11)	.65	.26 - 1.60
**Circumcision^e^**								
No	469(93)	98(20.90)	1.0		469(93)	61(13.01)	1.0	
Yes	35(7)	5(14.29)	.61	.22 - 1.64	35(7)	8(22.86)	1.92	.82 - 4.51
**History of sexual relations ** **with prostitutes**								
No	395(78.37)	72(18.23)	1.0		--	--	--	
Yes	109(21.63)	31(28.44)	1.68	1.01 - 2.78	--	--	--	--
**Use of condom when having sexual ** **relations with prostitutes**								
Have not had sexual relations with prostitutes	395(78.37)	72(18.23)	1.0		--	--	--	
Always	34(6.75)	8(23.53)	1.46	.63 - 3.41	--	--	--	--
Not always	75(14.88)	23(30.67)	1.78	1.004 - 3.17	--	--	--	--
*P-trend*				0.0128				

^a ^Due to missing data, all categories do not total 504.

^b^Odds ratio and 95% confidence intervals obtained using logistic regression models adjusted for age and SLI.

^c ^Models adjusted for SLI only to avoid colinearity.

^d ^Models adjusted for age only to avoid colinearity when adjusting for SLI.

^e ^This variable as was asked of men only. Women were assigned the value corresponding to the antecedent of circumcision in their male sexual partner.

#### Sexual behavior characteristics associated with the presence of HPV infection

Forty-eight percent of the men reported having three or more lifetime sexual partners; Compared with 9% among women. The median number of sexual intercourses per month among the partners was eight, 29% of partners reported having sexual relations between 11 and 30 times a month. Thirty-seven percent of the men and 33% of the women reported having had anal sexual relations. Circumcision was confirmed in 7% of the male participants. Among men, initiating active sexual life before the age of 18 was positively associated with current penile HPV infection (OR 1.6, 95%CI 1.0 - 2.5) as was having had 10 or more lifetime sexual partners (OR 2.5, 95%CI 1.3 - 4.8), a history of having had sexual relations with prostitutes (OR 1.7, 95%CI 1.01 - 2.8) ****and not using condoms on a regular basis when having relations with prostitutes (OR 1.8, 95%CI 1.0 - 3.2) (Table 3). The percentage of men reporting having had two or more current regular sexual partners was 13.7%, 44.6% reported having had sexual intercourse with occasional partners, 21.6% with prostitutes, and 13.9% had maintained sexual relations with prostitutes while maintaining sexual relations with their regular partner (data not shown).

Among women, 4% had never been pregnant, 37.3% never had a cervical cytology, and 29.4% did not use any contraceptive method. An increased risk of cervical HPV infection was observed (OR 1.9, 95%CI 1.01 - 3.7) in women whose male partners had sexual relations with prostitutes while living together, as was for those whose partners did not use condoms while having relations with prostitutes (OR 1.9, 95%CI 1.0 - 3.6). In women, no statistically significant associations were found between sexual behavior characteristics and HPV detection (data not shown).

The presence of any HPV type infection in men was strongly associated with the presence of HPV infection in their female sexual partners (OR 5.1, 95%CI 3.0 - 8.8) (Table 4).

**Table 4:** Risk of HPV infection associated with the status of HPV infection in the sexual partner.

Variable	Risk of HPV infection in women				
	
*Presence of HPV in men*	n = 504	HPV positives n = 69			
		%	OR^a^	ρ^a^	CI 95%^a^
**Presence of HPV**					
Negative	401/79.56	8.73(35)			
Positive	103/20.44	33.01(34)	5.15	0.000	3.01 - 8.82
**Presence of oncogenic HPV**					
Negative	460	6.96(32)			
Positive	44	36.36(16)	7.64	0.000	3.75 - 15.56
**Presence of nononcogenic HPV**					
Negative	429	3.73(16)			
Positive	75	22.67(17)	7.56	0.000	3.62 - 15.79
**Presence of HPV**					
16 and/or 18					
Negative	491	2.44(12)			
Positive	13	15.38(2)	7.25	0.016	1.44 - 36.37

^a^Odds ratio, p-value, and CI 95% obtained using logistic regression.

## Conclusions

This work describes one of the first studies in a Mexican population that evaluates HPV type-specific concordance among heterosexual couples in a rural area in central Mexico. Of 138 couples where at least one partner was infected, approximately 25% (34/138 = 24.6) of the respective partners were simultaneously infected by HPV. Among these couples, type-specific concordance was high (61.8%). The principal predictors of HPV in men were factors related to high-risk sexual behavior. The presence of HPV in both men and women was strongly associated with the detection of HPV in their respective partners.

Studies over the past 20 years evaluating HPV infection concordance among heterosexual partners have shown many inconsistencies, reporting concordances of type-specific infection of between 2 and 87%[5,6,8,16-21]. Such heterogeneous findings may be due to the use of diverse laboratory HPV DNA detection techniques, the methods used to select the study population, and to the anatomical site being sampled (particularly in men), among other factors.

An early report on HPV concordance in heterosexual partners documented that 75% of women whose male partners had HPV were also HPV positive, while only 39% of men with HPV-positive female partners were HPV positive in semen[22]. Female partners of men with condylomatosis of the penis have also been studied, where high-risk cervical HPV has been calculated to be 27.7% and cytologic anomalies in the cervix has been estimated to be 36%[23]. The main limitation of previous studies was primarily methodological. Technological developments over the years in the area of diagnostic testing have led to more sensitive HPV DNA detection tests. Furthermore, the identification of male anatomical regions where HPV is routinely detected has recently been well studied[12]. Therefore, comparisons between population studies are greatly limited due to differences in the methods employed.

Other studies have recently shown concordance findings to be similar to those found in this study; 76% of male partners of infected women have been shown to be HPV positive[18]. Three other studies evaluating type-specific concordance in heterosexual partners reported concordance estimates of 43% a 64.4%, although the sample size was quite small[8,19,24]. These results are consistent with the hypothesis of sexual transmission[25,26] of HPV infections.

An association between the presence of lesions in the sexual partner and the presence of HPV infection was not demonstrated in the current study as the large proportion of infections in this population was subclinical. It is possible that the combined sampling of sites of the scrotum and penile shaft, balano-preputial lamina and urinary meatus has increased the type-specific concordance value found in this study. It has been shown in a previous study that use of combined samples increases HPV DNA detection[27].

The 13.7% prevalence of HPV infection found in women is less than the prevalence of 20.4% found in men; this lower prevalence in women compared to their male partners has been observed in other studies that evaluate both men and women[27]. The natural history of HPV infection may be different between men and women due to differences between the epithelium in the cervical transformation zone and the penis. HPV DNA prevalence has been shown to be as much as two to three times higher in Mexican men than in Mexican women. With respect to women, HPV prevalence in Mexico has been reported as ranging from as little as 3.7% to as high as 48.9%[28-33] and a systematic review conducted in the United States that includes studies of prevalence in Hispanic, African-American, Asian, Caucasian and other women report prevalences between 14% and 90%[34]. Higher HPV prevalence estimates have been observed among women with high-risk sexual behavior as compared to predominantly monogamous women[35]. This is consistent with prevalence estimates derived from a population-based study of Mexican women and those from a study of women with social security health care services[30,32]. In addition, prevalence estimates in urban areas, where HPV is endemic, are greater than those observed among women in rural areas[36] and than those observed in countries with a low incidence of and mortality from cervical cancer[37]. The bimodal pattern for HPV infection by age group observed in previous studies of populations with elevated mortality due to cervical cancer[28,37] was not observed in this study of rural women, showing an elevated prevalence of more than 14% in women older than 30 years, which was consistent up to 75 years of age, the maximum age included.

The above is a reflection of the fact that HPV prevalence of and concordance among couples is not only highly variable but also depends on the sexual behavior of the couples, the sensitivity of the tests employed, and more importantly on the differences between acquisition rates among men and women.

The identification of risk factors associated with HPV detection in our study in both men and women is consistent with that of other reports[38-48]. In men, being single and having fewer years of schooling are associated with an increased risk for HPV infection, as is being younger on sexual debut, having multiple sexual partners, and a history of having had sexual relations with prostitutes. This pattern of risk factors for penile HPV infection is similar to that found in an HPV study among Mexican soldiers[40]. For women, being single and smoking are factors clearly correlated with high-risk sexual conducts and are therefore positively associated with HPV infection, a finding that is also consistent with reports from previous studies[32,37]. In the present study, we show for Mexican men that a history of sexual relations with sex workers and inadequate use of condoms when having such sexual relations increase the risk of HPV infection in their female partners, indicating, as in many other studies, the key role of the male factor in the risk of HPV infection in the female partner.

The results of this study of couples are singularly important, in part, because this is one of the first studies to quantify type-specific HPV concordance for a female population with a pattern of sexual conduct that is predominantly monogamous. In addition, because HPV prevalence estimates in the external male genitalia was found to be two times higher than that previously reported for Mexican men with low-risk sexual behavior. These findings are bolstered by an external quality control mechanism for the determination of HPV DNA in the study population that included a determination of HPV blind to knowledge of gender and of the HPV results of the corresponding partner. Therefore, information bias with regard to the characterization of the presence of HPV DNA is improbable.

Prospective cohort studies among different populations are warranted to confirm these estimates as well as to quantify the probability of HPV transmission patterns in men and women and explore the role of potentially associated cofactors.

## Competing interests

The authors declare that they have no competing interests.

## Authors' contributions

RP participated in the design of the study, supervised the trial and data acquisition process, RM performed the statistical analysis, interpreted the data and wrote this paper, ARG and XC analyzed and interpreted the data, AC participated in the design of the study, ELP conceived of the study, participated in its design, coordination and wrote this paper. All authors read and approved the final manuscript.

## Pre-publication history

The pre-publication history for this paper can be accessed here:

http://www.biomedcentral.com/1471-2334/10/223/prepub

## References

[B1] Bosch FX Castellsague X Munoz N de Sanjose S Ghaffari AM Gonzalez LC Gili M Izarzugaza I Viladiu P Navarro C Vergara A Ascunce N Guerrero E Shah KV Male sexual behavior and human papillomavirus DNA: key risk factors for cervical cancer in Spain J Natl Cancer Inst 1996 88 1060 1067 10.1093/jnci/88.15.1060 8683637

[B2] Zunzunegui MV King MC Coria CF Charlet J Male influences on cervical cancer risk Am J Epidemiol 1986 123 302 307 3753819 10.1093/oxfordjournals.aje.a114238

[B3] Taner MZ Taskiran C Onan MA Uluturk A Himmetoglu O Genital human papillomavirus infection in the male sexual partners of women with isolated vulvar lesions Int J Gynecol Cancer 2006 16 791 794 10.1111/j.1525-1438.2006.00376.x 16681762

[B4] Rombaldi RL Serafini EP Villa LL Vanni AC Barea F Frassini R Xavier M Paesi S Infection with human papillomaviruses of sexual partners of women having cervical intraepithelial neoplasia Braz J Med Biol Res 2006 39 177 187 10.1590/S0100-879X2006000200003 16470304

[B5] Castellsagué X Ghaffari A Daniel RW Bosch FX Muñoz N Shah KV Prevalence of penile human papillomavirus DNA in husbands of women with and without cervical neoplasia: a study in Spain and Colombia J Infect Dis 1997 176 353 361 10.1086/514052 9237700

[B6] Franceschi S Castellsagué X Dal Maso L Smith JS Plummer M Ngelangel C Chichareon S Eluf-Neto J Shah KV Snijders PJ Meijer CJ Bosch FX Muñoz N Prevalence and determinants of human papillomavirus genital infection in men Br J Cancer 2002 86 705 711 10.1038/sj.bjc.6600194 11875730 PMC2375316

[B7] Bleeker MC Hogewoning CJ Voorhorst FJ van den Brule AJ Berkhof J Hesselink AT Lettink M Starink TM Stoof TJ Snijders PJ Meijer CJ HPV-associated flat penile lesions in men of a non-STD hospital population: less frequent and smaller in size than in male sexual partners of women with CIN Int J Cancer 2005 113 36 41 10.1002/ijc.20502 15386360

[B8] Bleeker MC Hogewoning CJ Berkhof J Voorhorst FJ Hesselink AT van Diemen PM van den Brule AJ Snijders PJ Meijer CJ Concordance of specific human papillomavirus types in sex partners is more prevalent than would be expected by chance and is associated with increased viral loads Clin Infect Dis 2005 41 612 620 10.1086/431978 16080082

[B9] Bleeker MC Berkhof J Hogewoning CJ Voorhorst FJ van den Brule AJ Starink TM Snijders PJ Meijer CJ HPV type concordance in sexual couples determines the effect of condoms on regression of flat penile lesions Br J Cancer 2005 92 1388 1392 10.1038/sj.bjc.6602524 15812547 PMC2361997

[B10] Winer RL Feng Q Hughes JP O'Reilly S Kiviat NB Koutsky LA Risk of female human papillomavirus acquisition associated with first male sex partner J Infect Dis 2008 197 279 282 10.1086/524875 18179386 PMC2875685

[B11] Burchell AN Richardson H Mahmud SM Trottier H Tellier PP Hanley J Coutlee F Franco EL Modeling the sexual transmissibility of human papillomavirus infection using stochastic computer simulation and empirical data from a cohort study of young women in Montreal, Canada Am J Epidemiol 2006 163 534 543 10.1093/aje/kwj077 16421235

[B12] Aguilar LV Lazcano-Ponce E Vaccarella S Cruz A Hernández P Smith JS Muñoz N Kornegay JR Hernández-Avila M Franceschi S Human papillomavirus in men: comparison of different genital sites Sex Transm Infect 2006 82 31 33 10.1136/sti.2005.015131 16461598 PMC2563819

[B13] Sambrook J Fitsch EF Maniatis T Molecular Cloning: A Laboratory Manual 1989 Cold Spring Harbor, Cold Spring Harbor Press

[B14] Gravitt PE Peyton CL Apple RJ Wheeler CM Genotyping of 27 human papillomavirus types by using L1 consensus PCR products by single hybridization, reverse line blot detection method J Clin Microbiol 1998 36 3020 3027 9738060 10.1128/jcm.36.10.3020-3027.1998 PMC105104

[B15] Cogliano V Baan R Straif K Grosse Y Secretan B El Ghissassi F Carcinogenicity of human papillomaviruses Lancet Oncol 2005 6 204 10.1016/S1470-2045(05)70086-3 15830458

[B16] Rosenblatt C Lucon AM Pereyra EA Pinotti JA Arap S Ruiz CA HPV prevalence among partners of women with cervical intraepithelial neoplasia Int J Gynaecol Obstet 2004 84 156 161 10.1016/j.ijgo.2003.08.008 14871518

[B17] Hippelainen MI Yliskoski M Syrjanen S Saastamoinen J Hippeläinen M Saarikoski S Syrjänen K Low concordance of genital human papillomavirus (HPV) lesions and viral types in HPV-infected women and their male sexual partners Sex Transm Dis 1994 21 76 82 10.1097/00007435-199403000-00004 9071416

[B18] Baken LA Koutsky LA Kuypers J Kosorok MR Lee SK Kiviat NB Holmes KK Genital human papillomavirus infection among male and female sex partners: prevalence and type-specific concordance J Infect Dis 1995 171 429 432 7844382 10.1093/infdis/171.2.429

[B19] Benevolo M Mottolese M Marandino F Carosi M Diodoro MG Sentinelli S Visca P Rollo F Mariani L Vocaturo G Sindico R Terrenato I Donnorso RP Vocaturo A HPV prevalence among healthy Italian male sexual partners of women with cervical HPV infection J Med Virol 2008 80 1275 1281 10.1002/jmv.21189 18461608

[B20] Nicolau SM Camargo CG Stavale JN Castelo A Dôres GB Lörincz A de Lima GR Human papillomavirus DNA detection in male sexual partners of women with genital human papillomavirus infection Urology 2005 65 251 255 10.1016/j.urology.2004.09.031 15708032

[B21] Strand A Rylander E Wilander E Zehbe I HPV infection in male partners of women with squamous intraepithelial neoplasia and/or high-risk HPV Acta Derm Venereol 1995 75 312 316 8578958 10.2340/0001555575312316

[B22] Kyo S Inoue M Koyama M Fujita M Tanizawa O Hakura A Detection of high-risk human papillomavirus in the cervix and semen of sex partners J Infect Dis 1994 170 682 685 8077728 10.1093/infdis/170.3.682

[B23] Campion MJ Singer A Clarkson PK McCance DJ Increased risk of cervical neoplasia in consorts of men with penile condylomata acuminata Lancet 1985 1 943 946 10.1016/S0140-6736(85)91724-6 2859410

[B24] Giovannelli L Bellavia C Capra G Migliore MC Caleca M Giglio M Perino A Matranga D Ammatuna P HPV group- and type-specific concordance in HPV infected sexual couples J Med Virol 2007 79 1882 1888 10.1002/jmv.21015 17935193

[B25] Rylander E Ruusuvaara L Almströmer MW Evander M Wadell G The absence of vaginal human papillomavirus 16 DNA in women who have not experienced sexual intercourse Obstet Gynecol 1994 83 735 737 8164934

[B26] Kjaer SK Chackerian B van den Brule AJ Svare EI Paull G Walbomers JM Schiller JT Bock JE Sherman ME Lowy DR Meijer CL High-risk human papillomavirus is sexually transmitted: evidence from a follow-up study of virgins starting sexual activity (intercourse) Cancer Epidemiol Biomarkers Prev 2001 10 101 106 11219765

[B27] Dunne EF Nielson CM Stone KM Markowitz LE Giuliano AR Prevalence of HPV infection among men: A systematic review of the literature J Infect Dis 2006 194 1044 1057 10.1086/507432 16991079

[B28] Lazcano-Ponce E Herrero R Munoz N Cruz A Shah KV Alonso P Hernandez P Salmeron J Hernandez M Epidemiology of HPV infection among Mexican women with normal cervical cytology Int J Cancer 2001 91 412 420 10.1002/1097-0215(20010201)91:3<412::AID-IJC1071>3.0.CO;2-M 11169968

[B29] Hernández-Girón C Smith JS Lorincz A Arreola Cháidez E Lazcano E Hernández-Avila M Salmerón J The prevalence of high-risk HPV infection in pregnant women from Morelos, México Salud Publica Mex 2005 47 423 429 16983987 10.1590/s0036-36342005000600006

[B30] Hernández-Girón C Smith JS Lorincz A Lazcano E Hernández-Avila M Salmerón J High-risk human papillomavirus detection and related risk factors among pregnant and nonpregnant women in Mexico Sex Transm Dis 2005 32 613 618 10.1097/01.olq.0000179888.47309.db 16205302

[B31] Sánchez-Alemán MA Uribe-Salas F Conde-González CJ La infección por el virus del Papiloma humano, un posible marcador biológico del comportamiento sexual en estudiantes universitarios Salud Publica Mex 2002 44 442 447 12389488

[B32] Juárez-Figueroa LA Wheeler CM Uribe-Salas FJ Conde-Glez CJ Zampilpa-Mejía LG García-Cisneros S Hernández-Avila M Human papillomavirus: a highly prevalent sexually transmitted disease agent among female sex workers from Mexico City Sex Transm Dis 2001 28 125 130 10.1097/00007435-200103000-00001 11289192

[B33] Rodríguez-Reyes ER Quiñónez-Pérez JM Cerda-Flores RM Saucedo-Cardenas O Cortés-Gutiérrez EI Prevalencia del VPH en sexoservidoras de Durango, México Salud Publica Mex 2005 47 393 16983982 10.1590/s0036-36342005000600001

[B34] Revzina NV Diclemente RJ Prevalence and incidence of human papillomavirus infection in women in the USA: a systematic review Int J STD AIDS 2005 16 528 537 10.1258/0956462054679214 16105186

[B35] Clifford GM Gallus S Herrero R Muñoz N Snijders PJ Vaccarella S Anh PT Ferreccio C Hieu NT Worldwide distribution of human papillomavirus types in cytologically normal women in the International Agency for Research on Cancer HPV prevalence surveys: a pooled analysis Lancet 2005 366 991 998 10.1016/S0140-6736(05)67069-9 16168781

[B36] Garcia PJ Chavez S Feringa B Chiappe M Li W Jansen KU Carcamo C Holmes KK Reproductive tract infections in rural women from the highlands, jungle, and coastal regions of Peru Bull World Health Organ 2004 82 483 492 15508193 PMC2622905

[B37] De Sanjosé S Diaz M Castellsagué X Clifford G Bruni L Muñoz N Bosch FX Worldwide prevalence and genotype distribution of cervical human papillomavirus DNA in women with normal cytology: a meta-analysis Lancet Infect Dis 2007 7 453 459 10.1016/S1473-3099(07)70158-5 17597569

[B38] Vaccarella S Lazcano-Ponce E Castro-Garduno JA Cruz-Valdez A Diaz V Schiavon R Hernandez P Kornegay JR Hernandez-Avila M Franceschi S Prevalence and determinants of human papillomavirus infection in men attending vasectomy clinics in Mexico Int J Cancer 2006 119 1934 1939 10.1002/ijc.21992 16708372

[B39] Lazcano-Ponce E Herrero R Muñoz N Hernandez-Avila M Salmerón J Leyva A Meijer CJ Walboomers JM High prevalence of human papillomavirus infection in Mexican males: comparative study of penile-urethral swabs and urine samples Sex Transm Dis 2001 28 277 280 10.1097/00007435-200105000-00007 11354266

[B40] Lajous M Mueller N Cruz-Valdez A Aguilar LV Franceschi S Hernandez-Avila M Lazcano-Ponce E Determinants of prevalence, acquisition, and persistence of human papillomavirus in healthy Mexican military men Cancer Epidemiol Biomarkers Prev 2005 14 1710 1716 10.1158/1055-9965.EPI-04-0926 16030106

[B41] Kjaer SK Munk C Winther JF Jørgensen HO Meijer CJ van den Brule AJ Acquisition and persistence of human papillomavirus infection in younger men: a prospective follow-up study among Danish soldiers Cancer Epidemiol Biomarkers Prev 2005 14 1528 1533 10.1158/1055-9965.EPI-04-0754 15941967

[B42] Vaccarella S Lazcano-Ponce E Castro-Garduño JA Cruz-Valdez A Díaz V Schiavon R Hernández P Kornegay JR Hernández-Avila M Franceschi S Prevalence and determinants of human papillomavirus infection in men attending vasectomy clinics in Mexico Int J Cancer 2006 119 1934 1939 10.1002/ijc.21992 16708372

[B43] Partridge JM Hughes JP Feng Q Winer RL Weaver BA Xi LF Stern ME Lee SK O'Reilly SF Hawes SE Kiviat NB Koutsky LA Genital human papillomavirus infection in men: incidence and risk factors in a cohort of university students J Infect Dis 2007 196 1117 1119 10.1086/521192 17955430

[B44] Giuliano AR Papenfuss M Schneider A Nour M Hatch K Risk factors for high-risk type human papillomavirus infection among Mexican-American women Cancer Epidemiol Biomarkers Prev 1999 8 615 620 10428199

[B45] Bauer HM Hildesheim A Schiffman MH Glass AG Rush BB Scott DR Cadell DM Kurman RJ Manos MM Determinants of genital human papillomavirus infection in low-risk women in Portland, Oregon Sex Transm Dis 1993 20 274 278 10.1097/00007435-199309000-00007 8235925

[B46] Sellors JW Mahony JB Kaczorowski J Lytwyn A Bangura H Chong S Lorincz A Dalby DM Janjusevic V Keller JL Prevalence and predictors of human papillomavirus infection in women in Ontario, Canada. Survey of HPV in Ontario Women (SHOW) Group CMAJ 2000 163 503 508 11006760 PMC80454

[B47] Lazcano-Ponce E Herrero R Muñoz N Cruz A Shah KV Alonso P Hernández P Salmerón J Hernández M Epidemiology of HPV infection among Mexican women with normal cervical cytology Int J Cancer 2001 91 412 420 10.1002/1097-0215(20010201)91:3<412::AID-IJC1071>3.0.CO;2-M 11169968

[B48] Rousseau MC Abrahamowicz M Villa LL Costa MC Rohan TE Franco EL Predictors of cervical coinfection with multiple human papillomavirus types Cancer Epidemiol Biomarkers Prev 2003 12 1029 1037 14578139

